# 988. Clinical Validation of a Novel Point of Care Mp1p Antigen Lateral Flow Immunoassay to Diagnose *Talaromyces marneffei* Infection

**DOI:** 10.1093/ofid/ofad500.043

**Published:** 2023-11-27

**Authors:** Sruthi Venugopalan, Thu Nguyen, Konner Bloss, B S Chemistry, Helen Xu, Vo Trieu Ly, Ngo Thi Hoa, Jianpiao Cai, Jasper F W Chan, Kwok-Yung Yuen, Thuy Le

**Affiliations:** Duke University School of Medicine, Durham, NC; Duke University School of Medicine, Durham, NC; IMMY, Norman, Oklahoma; Duke University, Durham, North Carolina; University of medicine and pharmacy at Ho Chi Minh city, Vietnam, Ho Chi Minh, Ho Chi Minh, Vietnam; OUCRU, Ho Chi Minh City, Ho Chi Minh, Vietnam; Department of Microbiology, The University of Hong Kong, Hong Kong, Not Applicable, Hong Kong; The University of Hong Kong, Hong Kong, Not Applicable, Hong Kong; The University of Hong Kong, Hong Kong, Not Applicable, Hong Kong; Duke University School of Medicine, Durham, NC

## Abstract

**Background:**

Talaromycosis, an invasive mycosis caused by the Southeast Asian fungus *Talaromyces marneffei* (Tm) is a leading cause of AIDS-associated death in this region. Lack of non-culture diagnostics is the greatest hurdle to reducing mortality. We have previously developed a novel Mp1p antigen enzyme immunoassay (EIA) showing superior sensitivity compared to blood culture. Here, we report the clinical validation of the Mp1p lateral flow assay (LFA) developed by IMMY in patient cohorts.

**Methods:**

We employed a nested case control cohort study using paired plasma and urine samples from two prospective cohorts in Vietnam. Cases (N=132) were hospitalized patients with advanced HIV disease and culture-proven talaromycosis. Controls (N=160) were randomly-selected patients who did not develop culture-proven talaromycosis over 6-month follow up (**Fig. 1**). The procedures for the Mp1p LFA were shown in **Fig. 2**. We constructed the ROC curves and compared the sensitivity and specificity of the Mp1p LFA vs EIA against the culture-confirmed talaromycosis reference standard using McNemar tests. The results of the LFA were interpreted by 3 double-blinded readers and interrater reliability (IRR) was determined by Cohen’s kappa.

Study population

**Figure:**
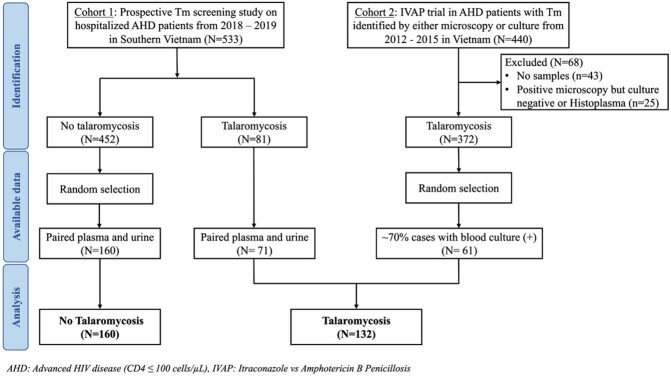

Procedure of the Mp1p LFA

**Figure:**
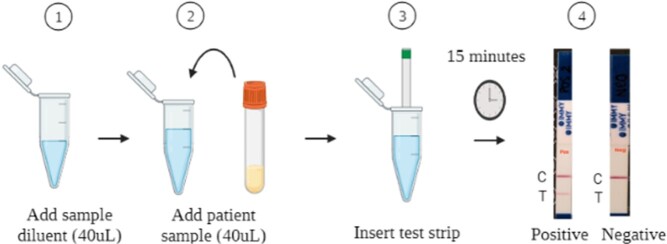

Add 40µL of specimen diluent buffer and 40µL of thawed patient sample to a flat bottom microcentrifuge tube and mix well. Insert test strip (IMMY) into tube and read results after 15 minutes. The strip has two lines, 'C' for control and 'T’ for test line. The presence of 2 lines in both 'C' and 'T' indicates a positive test. The presence of a line only in 'C' indicates a negative test. The presence of a line only in 'T' indicates an invalid test.

**Results:**

Diagnostic performances of the Mp1p LFA and EIA were similar (**Fig. 3**). In plasma: sensitivity 82% vs 84%, *P* = 0.25; specificity 99% vs 98%, *P* = 0.37. In urine: sensitivity 90% vs 92%, *P* = 0.25; specificity: 99% vs 100%, *P* = 1.00. Sensitivities were significantly higher in urine compared to plasma for both the Mp1p LFA (90% vs 82%, *P* = 0.02) and Mp1p EIA (92% vs 84%, *P* = 0.02); while specificity was similar (**Fig. 4**). The sensitivity improved further when testing in plasma and urine in combination compared to testing in plasma alone for the Mp1p LFA (93% vs 82%, *P* < 0.001) and for the Mp1p EIA (95% vs 84%, *P* < 0.001). The IRR between 3 blinded readers was in almost perfect agreement (kappa=0.89, *P = 0*).

Comparative diagnostic performance of the Mp1p LFA vs Mp1p EIA when performed in plasma samples (A), in urine samples (B), and in plasma and urine samples concurrently (C)

**Figure:**
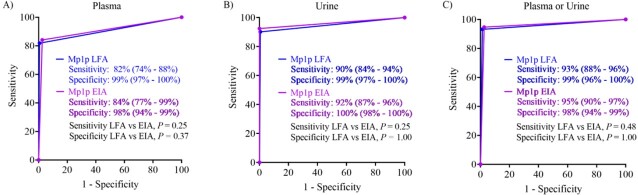

Comparative diagnostic performance of the Mp1p LFA and Mp1p EIA when performed in plasma vs urine samples

**Figure:**
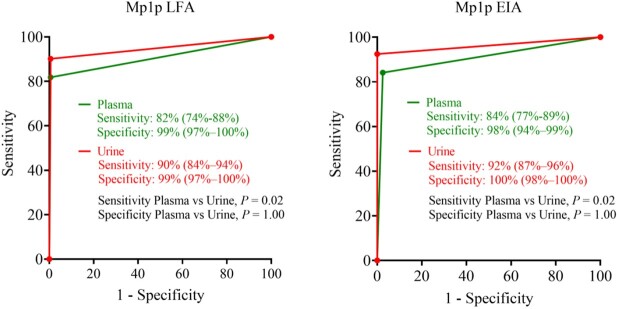

The receiver operating characteristic (ROC) curve demonstrates that urine had significantly higher sensitivity compared to plasma for both assays (90% vs 82% for the Mp1p LFA [P = 0.02, McNemar test]); 92% vs 84% for the Mp1p EIA [P = 0.02, McNemar test]); while specificity was similar.

**Conclusion:**

We observed similar performance between the Mp1p LFA and EIA. Sensitivity was higher in urine vs plasma in both assays, and was higher when testing plasma and urine concurrently. The Mp1p LFA is easy to perform and satisfies the ‘ASSURED’ criteria by WHO for a point of care diagnostic test and has potential to change the management of talaromycosis.

**Disclosures:**

**Jasper FW Chan, MBBS(HK), MD(HK), FIDSA**, IMMY: Development of Talaromyces diagnostics

